# Suppression of Abdominal Motor Activity during Swallowing in Cats and Humans

**DOI:** 10.1371/journal.pone.0128245

**Published:** 2015-05-28

**Authors:** Teresa Pitts, Albright G. Gayagoy, Melanie J. Rose, Ivan Poliacek, Jillian A. Condrey, M. Nicholas Musslewhite, Tabitha Y. Shen, Paul W. Davenport, Donald C Bolser

**Affiliations:** 1 Department of Neurological Surgery, Kentucky Spinal Cord Injury Research Center, University of Louisville, Louisville, KY, United States of America; 2 Department of Physiological Sciences, University of Florida, Gainesville, FL, United States of America; Faculty of Animal Sciences and Food Engineering, University of São Paulo, Pirassununga, SP, Brazil, BRAZIL

## Abstract

Diseases affecting pulmonary mechanics often result in changes to the coordination of swallow and breathing. We hypothesize that during times of increased intrathoracic pressure, swallow suppresses ongoing expiratory drive to ensure bolus transport through the esophagus. To this end, we sought to determine the effects of swallow on abdominal electromyographic (EMG) activity during expiratory threshold loading in anesthetized cats and in awake-healthy adult humans. Expiratory threshold loads were applied to recruit abdominal motor activity during breathing, and swallow was triggered by infusion of water into the mouth. In both anesthetized cats and humans, expiratory cycles which contained swallows had a significant reduction in abdominal EMG activity, and a greater percentage of swallows were produced during inspiration and/or respiratory phase transitions. These results suggest that: a) spinal expiratory motor pathways play an important role in the execution of swallow, and b) a more complex mechanical relationship exists between breathing and swallow than has previously been envisioned.

## Introduction

The precise coordination of breathing and swallowing plays an important role to prevent entrance of food and other materials into the lower respiratory tract. Diseases which effect pulmonary mechanics such as chronic obstructive pulmonary disease (COPD), and or lung tumors result in changes to the coordination of swallow and breathing [1–3]. COPD is one of the thirteen statistically significant influencing factors that were implicated in the development of aspiration pneumonia [3–7]. Additionally, patients with COPD swallow more often during inspiration and consequently are at increased risk for post-swallow aspiration events [8, 9]. This disrupted breathing/swallow pattern could increase the risk of aspiration in patients with advanced COPD and may contribute to exacerbations [10, 11].

Expiratory threshold loading is an experimental technique which reliably elicits abdominal recruitment in human [12–20] and animal models [21–25]. A 15 cm H_2_0 expiratory threshold load increases rectus abdominis, internal oblique, transverses abdominis and gastric pressure to approximately 5–30% of maximum activity produced during cough [26]. Previous work [27] has increased our understanding of the interaction of swallow with other airway protective behaviors, and pressure threshold loading allows for the testing of its interaction with active expiration.

Pitts et al [28] proposed a dual valve system composed of highly coordinated control of both the laryngeal adductor and upper esophageal sphincter in regulating pressures between upper airway and the thoracic cavity, which controls the passage of air/bolus into or out of the lungs and esophagus. During swallowing, the pressure differential produced by upper esophageal relaxation and the maximal activity of tongue and pharyngeal muscles propels the bolus into the esophagus. During the expiratory phase of breathing, requirements for the production of swallowing are low because trans-laryngeal flows (i.e air movement through the larynx) and intra thoracic pressures are minimal. However, in patients with alterations in respiratory mechanics leading to increases in intra-thoracic pressure, such as with abdominal muscle recruitment, dysphagia may be promoted by hindrance of bolus movement across the upper esophageal sphincter.

The aims of this study were to determine if swallowing and breathing are coordinated during active expiration and if swallows affects pressure regulation by altering laryngeal and respiratory muscle activity. We hypothesized that swallows increase the duration and decrease the maximal activity of expiratory muscles during expiratory loading.

## Methods

### Animal Model

Approval for this study was granted from the University of Florida Institutional Animal Care and Use Committee (IACUC). The experiments were performed on six spontaneously breathing adult cats (5.2 ± 1.1 kg), obtained from Liberty Research, Inc, and housed at the University of Florida. The animals were anesthetized with sodium pentobarbital (35–40 mg/kg iv) and additional doses were given as needed (1–3mg/kg iv). The right femoral vein was cannulated for intravenous drugs administration and the right femoral artery was accessed for arterial blood sampling and blood pressure monitoring. A tracheostomy was performed and a cannula was inserted to allow spontaneous breathing. Arterial blood pressures, arterial blood gasses, end tidal CO_2_, and vital signs were monitored. An esophageal balloon was placed via an oral approach to measure pressure in the mid-thoracic esophagus. A rectal temperature probe was inserted to allow maintenance of body temperature at 37±1°C.

Electromyograms were recorded using bipolar insulated fine wire electrodes according to the technique of Basmajian and Stecko [29]. Seven muscles were used to evaluate breathing and swallowing: swallowing muscles (mylohyoid, geniohyoid, thyrohyoid, thyropharyngeus, and cricopharyngeus), inspiratory muscle (parasternal), and expiratory muscle (internal oblique). The muscles were identified through surgical dissection and visual inspection followed by electrode placement. The geniohyoid was exposed through a small incision on the rostral portion of the right mylohyoid. The thyroarytenoid electrodes were inserted through the cricothyroid window near the anterior portion of the vocal folds. The thyropharyngeus was spotted as a fan shaped muscle the wires are placed at the caudal portion at the thyroid cartilage attachment. At the posterior aspect of the larynx, the cricopharyngeus was identified and electrodes were placed just cranial to the edge of this structure. Thyrohyoid muscle electrodes were inserted rostral to its attachment to the thyroid cartilage. The parasternal muscle electrodes were placed on the third intercostal space adjacent to the sternum. Expiratory muscle electrodes were placed in the internal oblique muscle. The external oblique was moved without dissection to identify the internal oblique muscle. The positions of all electrodes were confirmed by electromyogram activity patterns during breathing and swallowing. Animals were euthanized by an overdose of sodium pentobarbital, followed by 3cc’s of potassium chloride.

### Protocol

A non-rebreathing valve was placed on the tracheal cannula. An expiratory threshold load of 15 cmH_2_0 was applied by attaching a hose to the expiratory port of this valve and immersing the end of the hose in a reservoir of water. Each load was 5 minutes in duration with the swallow trials beginning at the 2 minute mark. Swallow stimulation was completing by injecting 3 ml of water into the oropharynx via a syringe; this was repeated three times separated 1 minute. The swallows were identified from: a) a quiescence of the cricopharyngeus (UES), and b) overlapping large burst of activity of the mylohyoid, geniohyoid, thyropharyngeus, thyrohyoid, thyroarytenoid and the parasternal.

All EMG signals were amplified, filtered (200–5000 Hz), rectified, and integrated (time constant 50 ms). The inspiratory phase (T_I_), and the expiratory phase (T_E_) durations were measured. T_I_ was defined as the onset of parasternal activity to the maximum burst of the parasternal EMG, T_E_ was defined as the maximum burst of the parasternal EMG to the onset of the parasternal EMG activity for the next breath. The control (load-only) respiratory cycle durations (T_I_ and T_E_) were compared to cycles which contained a swallow. The maximum amplitude of the inspiratory muscles (parasternal) and expiratory muscles (internal oblique) were compared to cycles which contained a swallow.

### Human Model

Approval for this study was granted from the University of Florida Institutional Review Board (UF-IRB). All subjects provided written consent following the UF-IRB approved procedure. Five young (20 ± 1 years old) healthy males were recruited for this study. They had an average weight of 157 ± 42 pounds, height of 67 ± 3 inches, and a body mass index of 24 ± 7. All participants reported no history of swallow disorders, respiratory disease, and/or smoking within the last 10 years. The Institutional Review Board at the University of Florida approved the study.

Surface EMGs were affixed to the skin above the submental (including mylohyoid, geniohyoid and diagrastics) muscle group and the abdominal wall (lateral to the right rectus abdominis) over the oblique and transverses complex. To optimize abdominal recruitment all subjects were kneeling upright for the duration of the study. Subjects were asked to breathe through an apparatus comprised of a non-rebreathing valve, expiratory pressure threshold device (EMST 150, Aspire Products LLC), and a pressure transducer. The EMST 150 is a calibrated, one-way spring-loaded valve which has been used in studies for expiratory muscle strength training [12–19]. The pressure transducer confirmed the expiratory pressure necessary to overcome the load. A nose clip was affixed to ensure all airflow was through the oral cavity. During preliminary experiments, participants exposed to load significantly decreased their respiratory rate. For this set of experiments participants were asked to maintain their resting breathing rate during the loading paradigm, this decreased the need for larger expiratory threshold loads to record EMG abdominal recruitment.

### Protocol

The EMST150 was attached to the expiratory port of the non-rebreathing valve, placed into the subject’s mouth, and slowly the expiratory threshold level was increased until respiratory phasic abdominal EMG activity was observed (30–45 seconds in duration). Following this a 3cc syringe filled with sterile water with a one-inch tubing, was slid into the participants mouth (by the investigator) and slowly the water was infused over a 20 second time-period (0.15 ml/second). The subjects were instructed to “swallow when you feel it is necessary”. A five minute rest was given between each trial, and this was repeated 3 times.

For comparison all EMG amplitude measures are expressed as a percentage of the largest EMG amplitude. Results are expressed as means ± standard error. For statistical analysis Student’s paired *t*-tests were used to identify differences. A statistical difference was considered significant if the *p*-value was less than 0.05.

## Results

### Animal Model

**Fig 1:**
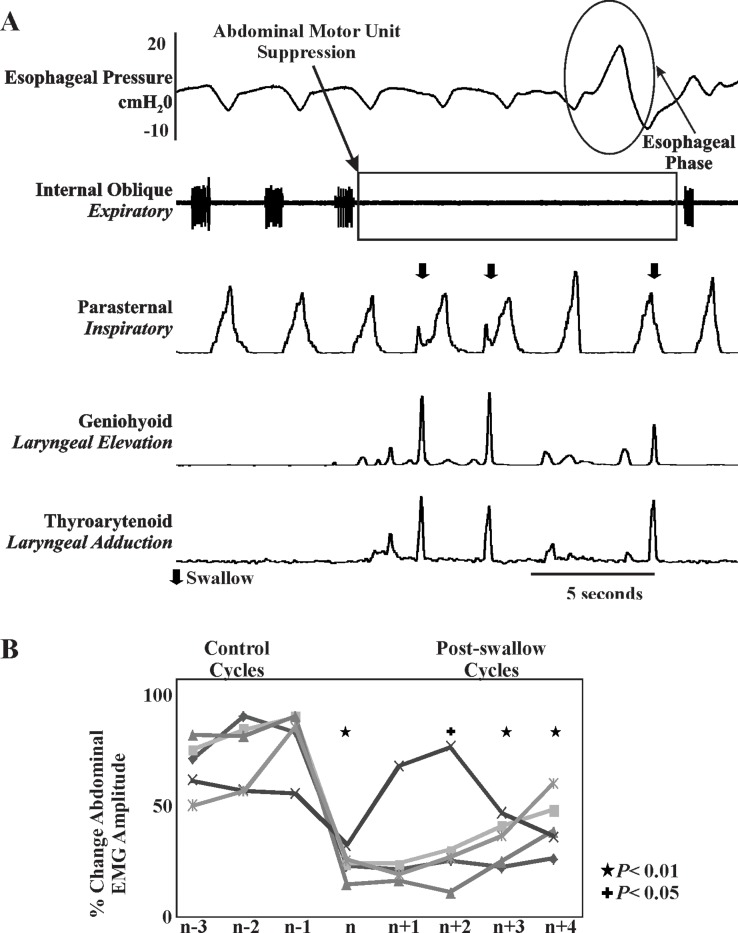
A. Example of abdominal motor unit suppression with swallow. Note the positive wave on the esophageal pressure channel. This is indicative of the peristaltic wave during the esophageal phase of swallow. Swallow is denoted by the arrow, the first 2 cycles occurred on the inspiratory-expiratory phase transition, the third is during the inspiratory phase of breathing. B. Line graph depicting average change in abdominal EMG amplitude for each of the five animals. N denotes the expiratory cycle that contained the swallow, n-1 to n-3 are the three preceding expiratory cycles, and n+1 to n+4 are the four following expiratory cycles. Four of the five animals had evidence of a multi-cycle suppression. For this analysis only single swallows which had 3 respiratory cycles preceding and following the swallow were included in this analysis.

The loading protocol recruited active abdominal activity in all animals (Fig 1). Fig 1a demonstrates an abdominal motor unit in the 3 respiratory cycles preceding a swallow and depression of the motor unit in the cycle containing a swallow and subsequent cycles. Note, for this analysis only single swallows which had 3 respiratory cycles preceding and following the swallow were included in this analysis, for a total of 25 swallows.

The maximum abdominal EMG activity in expiratory phase, which contained a swallow (55±12), was significantly lower that control expiratory cycles (95±2; *P* = 0.02); additionally, durations of T_E_ which contained a swallow (5.2±0.8 s) were significantly longer than those under the control condition (3.6±0.5 s; *P* = 0.04). The average time from the onset of the load to expiratory recruitment was 32 ± 13 s. Sixty percent (15 of 25) of swallows occurred during the expiratory phase with the remaining 40% (10 of 25) during inspiration. We did not observe swallows occurring during a respiratory phase. Fig 1b is a line graph demonstrating each animal’s averaged data for three cycles preceding the swallow and 4 cycles following the swallow.

The maximum parasternal EMG activity in inspiratory cycles, which contained a swallow (128±15), was higher but not significantly different to that during the inspiratory control cycles (94±2; *P* = 0.07). The duration of T_I_ which contained a swallow (1.2±0.3 s) was not significantly different than that under the control condition (1.0±0.08 s; *P* = 0.4) (Table 1).

**Table 1:** 

A. Respiratory EMG changes			
Amplitude (% Maximum)	Load	Load + Swallow	*P* value
*Cat*			
Parasternal	70 ± 7	93 ± 3	0.07
Rectus Abdominis	95 ± 1	45 ± 7	**<0.001**
*Human*			
Abdominal	90 ± 1	64 ± 8	**0.02**
Duration (ms)	Load	Load + Swallow	*P* value
*Cat*			
Inspiratory	961 ± 78	1225 ± 332	0.3
Expiratory	3667 ± 552	5184 ± 937	**0.04**
**B. Swallow EMG changes**			
Amplitude (% Maximum)	Rest Breathing	With Load	*P* value
*Cat*			
Mylohyoid	76 ± 8	73 ± 6	0.6
Geniohyoid	65 ± 20	76 ± 7	0.3
Thyrohyoid	76 ± 18	85 ± 4	0.4
Thyropharyngeus	63 ± 11	61 ± 16	0.7
Cricopharyngeus	59 ± 17	65 ± 19	0.7
Thyroarytenoid	74 ± 18	85 ± 6	0.2
Parasternal	47 ± 9	49 ± 11	0.5
*Human*			
Submental	73 ± 9	84 ± 3	0.3
*Significant *P*≤ 0.05			

**A**. Changes to inspiratory and expiratory EMG amplitude and duration comparing cycles with expiratory loading and expiratory loading with swallow. **B**. Changes to laryngeal, pharyngeal, and schluckatmung EMG amplitude during control swallows (rest breathing) and swallows during expiratory threshold loading.

There was no significant difference in the maximum EMG for swallows during the control versus the loading condition: mylohyoid (*P* = 0.6), geniohyoid (*P* = 0.3), thyrohyoid (*P* = 0.4), thyropharyngeus (*P* = 0.7), cricopharyngeus (*P* = 0.7), thyroarytenoid (*P* = 0.2), and parasternal (*P* = 0.5). All eighty swallows were included in this analysis, regardless of the respiratory phase in which they occurred.

### Human Model

**Fig 2:**
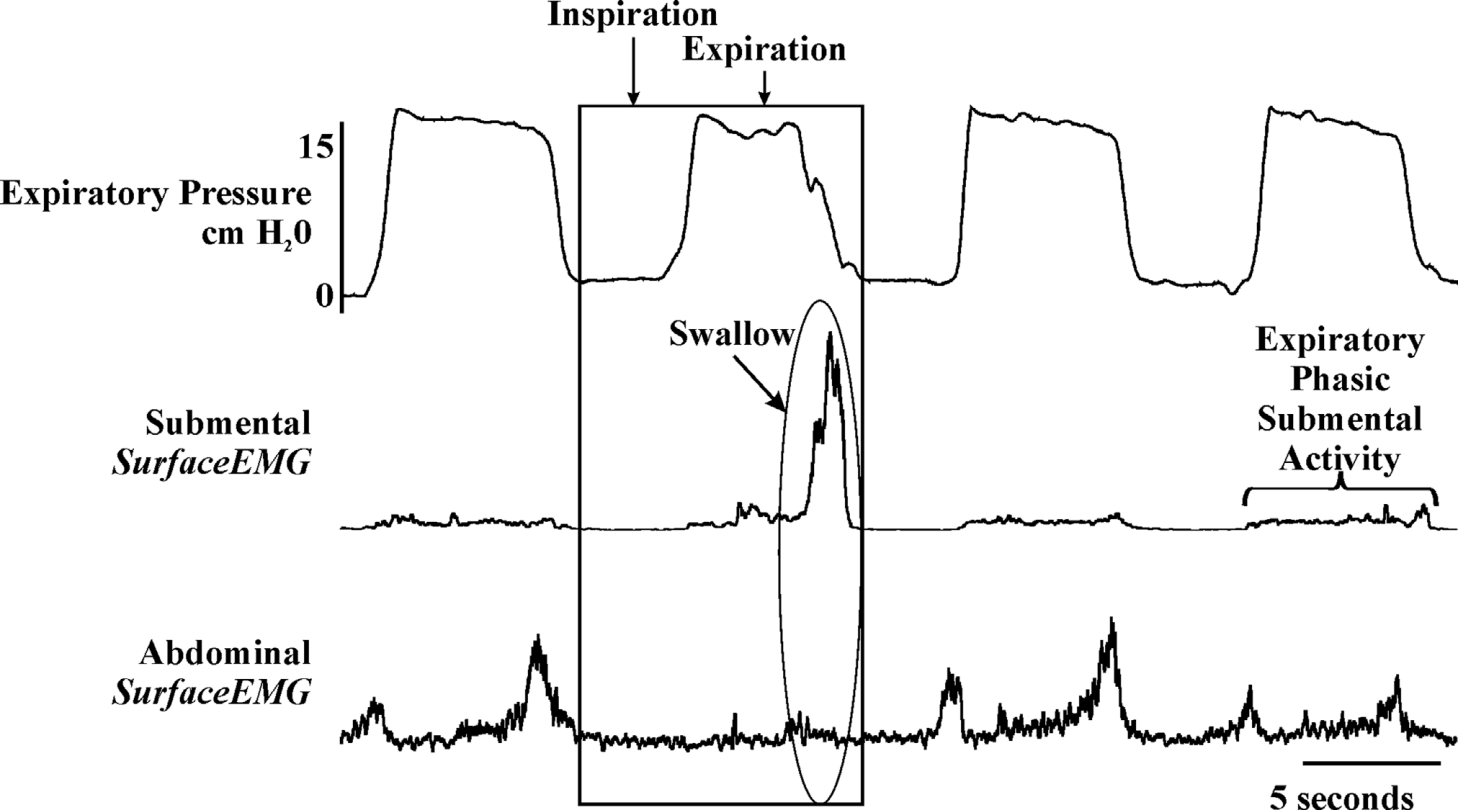
An example of swallowing in a young healthy male, and the abdominal suppression across the entire expiratory period. Note the small burst of abdominal activity at the beginning and the larger burst of abdominal activity at the end of the expiratory period was the consistent pattern.

The loading protocol recruited active abdominal activity in all subjects, which could be detected by surface EMG. The analysis included 54 swallows, and all subjects produced at least one swallow during the inspiratory phase of breathing. Fig 2 demonstrates abdominal suppression during an expiratory phase containing a swallow. Note the abdominal EMG burst, at the beginning of the expiratory cycle, is due to the active expiration required to open the pressure threshold load valve, to allow airflow. The maximum abdominal surface EMG activity of expiratory phases, which contained a swallow (64±8), was significant lower than that during the expiratory control phases (90±1; *P* = 0.02). The submental surface EMG amplitude (79±9) and duration was not significantly different for swallows during the loading condition (84±3; *P* = 0.29).

During the control condition 57 ± 11% of swallows occurred during the expiratory phase, with 5 ± 4% during the inspiration-expiratory transition, and 38±11% during the expiratory to inspiratory transition. However, during the loading condition 11 ± 9% of swallows occurred during the inspiratory phase, with a reduction of their occurrence during the expiratory phase (29 ± 14). Additionally 7 ± 6% of swallows occurred during the inspiratory-expiratory phase transition and the largest percentage (53 ± 15) occurred during the expiratory-inspiratory phase transition. Duration measurements were not obtained for the human measures. As noted previously, the subjects voluntarily constrained their respiratory frequency to the control rate. This instruction allowed for smaller expiratory threshold loads to be applied and increased subject compliance.

## Discussion

This is the first report of swallow suppressing the active abdominal recruitment during expiratory threshold loading in humans and cats. As seen in previous research: a) the expiratory threshold loading protocol increased abdominal EMG activity [14, 17, 21]; and shifted swallow from occurring predominately during expiration to inspiration [10, 11]. However, the present results demonstrate a more complex coordination system for breathing and swallow. Swallow has classically been thought of as a brainstem reflex that is produced solely by cranial and upper cervical motoneuron pools [30, 31]. Our results suggest that motoneuron pools in the thoracic and lumbar spinal cord also participate in the production of this behavior. Bautista, et al [32] in the perfused brainstem preparation of juvenile rats has also showed suppression of abdominal activity, however not as significant as was seen during the inspiratory phase of eupnoea. A limitation of the perfused brainstem preparation, is that there is no lung/thoracic phasic sensory feedback during breathing. Their findings, along with ours suggest that abdominal suppression by swallowing is a nascent feature of the core central pattern generator for swallow.

Both, our human subjects and experimental animals were unparalyzed, raising the possibility that peripheral feedback related to the mechanical changes that occur during a swallow could influence this larger depression in abdominal motor activity, seen in the present study. If so, the afferent sources likely arise from thoracic-abdominal somatic and/or visceral mechanoreceptors. A central source for these effects is also possible, consisting of depression of bulbospinal excitatory drive to abdominal motoneuron pools during swallow. Abdominal motoneuron pools receive their excitatory drive during breathing from expiratory phasic premotoneurons in the region of caudal nucleus retroamnbigualis, also known as the caudal ventral respiratory column [33].

Active expiratory suppression theoretically affected pressures across the upper esophageal sphincter to enhance bolus propulsion during swallowing. Negative intra-esophageal pressure during swallow has been described in humans [34–36] and animals [28, 37–40]; here referred to as the “schluckatmung” a German word meaning “swallow breath” [41–44]. McConnell has also published a series of papers describing this as the “hypopharyngeal suction pump” [45–59]. The source of this pressure has been disputed with two leading theories: a) phrenic nerve activity driving diaphragm activity [60, 61], or b) elevation of the laryngeal complex [46, 47, 58, 59]. It could also result from the combination of these two forces to create the necessary negative pressure. With the necessity of an adequate intra-esophageal pressure formation during swallow, now aligns swallow more with “inspiration during breathing” and the knowledge of lung mechanics can be applied.

During swallow, bolus movement is ensured by the combination of positive pressure (from the tongue/oral/velopharyngeal cavity), negative pressure (from the diaphragm and other accessory inspiratory muscles), along with pharyngeal squeezing which propels the bolus into the esophagus. However, even more important is the creation of a positive-negative pressure differential from the upper airway into the esophagus. Disorders such as COPD and lung cancer (large tumors) could create resting intra-thoracic pressures, with patient’s using active abdominal recruitment during the expiratory phase of breathing, which results in a mechanical disadvantage for execution of swallowing. This mechanical disadvantage could be overcome with multiple strategies including: suppression of ongoing active abdominal recruitment during swallows occurring during the expiratory phase of breathing, and/or shifting swallow phase to inspiration (as seen in these results), which would move the occurrence of swallow to the respiration phase with the greatest negative intrathoracic pressure.

A larger percentage of swallows were produced during inspiration or phase transitions in our experiments than are normally reported during control conditions [27, 39, 40, 62–65]. However, it is unknown whether the phase preference and phase shift during loading is a solely central/brainstem mediated phenomenon, or if continuous feedback from chest-wall, vagal, and/or abdominal afferents regulate swallow occurrence during active expiration. Additionally, during expiratory threshold loading there was no significant change in EMG amplitude in the laryngeal or pharyngeal muscles. This implies that pharyngeal activity is not necessarily subject to chestwall feedback; rather it is respiratory muscle motor control that is altered to promote bolus transfer. This is different than what was seen during the coordination of coughs and swallows in the cat; in which all pharyngeal and laryngeal muscle activity were significantly increased. This may represent a more delineated response to changes in respiratory status based on alterations in expiratory drive, which is significantly greater during cough than loading [21].

In summary, swallow breathing interactions are more complex than previously appreciated. Active expiratory suppression is an airway protective mechanism which should ensure than an adequate differential pressure is created across the upper esophageal sphincter. In conditions which modify respiratory mechanics, such as COPD and lung cancer, this may represent a significant strategy for maintaining the integrity of the bolus to ensure safe eating.
