# Correction: Suppression of Abdominal Motor Activity during Swallowing in Cats and Humans

**DOI:** 10.1371/journal.pone.0197525

**Published:** 2018-05-10

**Authors:** Teresa Pitts, Albright G. Gayagoy, Melanie J. Rose, Ivan Poliacek, Jillian A. Condrey, M. Nicholas Musselwhite, Tabitha Y. Shen, Paul W. Davenport, Donald C. Bolser

The sixth author’s name is incorrect. The correct name is: M. Nicholas Musselwhite. The correct citation is: Pitts T, Gayagoy AG, Rose MJ, Poliacek I, Condrey JA, Musselwhite MN, et al. (2015) Suppression of Abdominal Motor Activity during Swallowing in Cats and Humans. PLoS ONE 10(5): e0128245. https://doi.org/10.1371/journal.pone.0128245

## References

[1] Pitts T, Gayagoy AG, Rose MJ, Poliacek I, Condrey JA, Musslewhite MN, et al (2015) Suppression of Abdominal Motor Activity during Swallowing in Cats and Humans. PLoS ONE 10(5): e0128245 https://doi.org/10.1371/journal.pone.0128245 26020240 10.1371/journal.pone.0128245 PMC4447283

